# Conjugated Polyamines in Root Plasma Membrane Enhanced the Tolerance of Plum Seedling to Osmotic Stress by Stabilizing Membrane Structure and Therefore Elevating H^+^-ATPase Activity

**DOI:** 10.3389/fpls.2021.812360

**Published:** 2022-01-12

**Authors:** Hongyang Du, Benxue Chen, Qiang Li, Huaipan Liu, Ronald Kurtenbach

**Affiliations:** ^1^College of Life Science and Agronomy/Henan Key Laboratory of Crop Molecular Breeding and Bioreactor, Zhoukou Normal University, Zhoukou, China; ^2^College of Forestry, Beijing Forestry University, Beijing, China

**Keywords:** conjugated polyamines, H^+^-ATPase, osmotic stress, plum (*Prunus salicina* L.), plasma membrane

## Abstract

Polyamines are small positively charged molecules in plants and play important functions in many biological processes under various environmental stresses. One of the most confounding problems relating to polyamines (PAs) in stresses is the lack of understanding of the mechanisms underlying their function(s). Furthermore, a limited number of studies have addressed this issue at the sub-cellular level, especially in tree plants under drought stress. Therefore, in this research, by simulating natural drought stress with polyethylene glycol (PEG) osmotic stress, the relationship between the levels of conjugated polyamines and the activity of H^+^-ATPase in the plasma membrane was elucidated with the roots of two plum (*Prunus salicina* L.) cultivars, which were different in drought tolerance, as experimental materials. Furthermore, free PA levels and the activities of *S*-adenosylmethionine decarboxylase (SAMDC) and transglutaminase (TGase), which were closely associated with the levels of free and conjugated PAs, were also detected. Results showed that under osmotic stress, the increases of the levels of non-covalently conjugated (non-CC) spermidine (Spd) and spermine (Spm), covalently conjugated (CC) putrescine (Put) and Spd in the plasma membrane of drought-tolerant Ganli No. 5 were more significant than those of drought-sensitive Suli No. 3, indicating that these conjugated PAs might be involved in the tolerance of plum seedlings to stress. Furthermore, the conjugated PAs were closely correlated with plum seedling growth, water retention capacity, plasma membrane damage degree, and hydrogen (H^+^)-ATPase activity in the plasma membrane. To get more complementary pieces of evidence, we subjected plum seedlings to combined treatments of PEG and exogenous PA (Spd and Spm), and an inhibitor of SAMDC [methylglyoxal-bis (guanylhydrazone), (MGBG)] or TGase (o-phenanthroline). These results collectively suggested that non-CC Spd and Spm, CC Put and Spd in plasma membrane might function in enhancing the tolerance of plum seedlings to osmotic stress by stabilizing membrane structure and therefore elevating H^+^-ATPase activity.

## Introduction

Abiotic environmental factors, such as drought (Maheswari et al., 2016; Ouyang et al., 2020; Ebmeyer et al., 2021), temperature (Xu et al., 2018), heavy metal (Rakić et al., 2021), and salinity (Zheng et al., 2016; Zhong et al., 2020), are significant plant stressors with a major impact on plant development and productivity, thus causing serious agricultural yield losses. Among these stresses, drought is the most relevant (Maheswari et al., 2016; Ouyang et al., 2020; Ebmeyer et al., 2021). To respond to drought stress, plants have gradually evolved many sophisticated strategies on the different levels from changes in the related-gene expression to morphology alternations (Wujeska et al., 2013; Luo et al., 2020; Wang et al., 2020). Plum is one of the main forest fruit plants for human beings and in drought and semi-drought areas with little rain, plum seedlings are puniness and have to be frequently subjected to drought stress. So, the study on the plum tolerance to drought stress at the seedling stage is increasingly attractive, in which the changes in the levels of conjugated polyamine (PA) and the activity of hydrogen (H^+^)-ATPase in plasma membrane might be involved.

Multiple mechanisms contribute to plant adaptive responses to drought and each of these mechanisms is intrinsically dependent on membrane transporter activity, implying efficient regulation of the transporters under stress conditions. Furthermore, these transporters have long been considered as potential targets of reactive oxygen species, which are commonly increased in plants under a large number of abiotic stresses (including drought stress) (Zepeda-Jazo et al., 2011). As one of the important transporters in the plasma membrane, H^+^-ATPase operates as a powerhouse, controlling the electric potential difference, the active ion exchange across the plasma membrane (Palmgren, 2001), many cellular processes (Michelet et al., 1994; Chen et al., 2007), and thereby promoting cell dehydration tolerance. So, the operation of the plasma membrane H^+^-ATPase pump is central for responses to drought stress (Pottosin et al., 2021). It is well documented that under drought stress, plasma membrane H^+^-ATPase activity increases, and H^+^ secretion is enhanced, which results in the increased proton concentration gradient across the membrane. The proton concentration gradient provides a driving force for the secondary transport of extra-cellular osmotic regulatory substances (such as proline, glycine betaine, soluble sugar, *etc.*) into cells. The increases in the intracellular substances lead to a lower cell water potential, which is beneficial for cell absorbing water. For example, Gong et al. (2010) report that early activation of root hair cell H^+^-ATPase in the plasma membrane of drought-tolerant oat seedlings triggers the increases in major osmolytes, e.g., proline and glycine betaine, which lead to the up-regulation of the water maintenance system. For another example, the study of Cheng et al. (2021) demonstrate that arbuscular mycorrhizal fungus stimulates H^+^-ATPase activity in response to drought stress, which results in active physiological and biochemical processes, such as great nutrient uptake, root growth, photosynthetic and transpiration rate, *etc*., coupled with the enhanced tolerance of trifoliate orange seedlings to drought stress. Michelet et al. (1994) also indicate that the H^+^-ATPase activity in the plasma membrane is enhanced under drought conditions. Therefore, as an important protein, plasma membrane H^+^-ATPase is closely correlated to drought stress.

Polyamines (PAs) are unique polycationic metabolites and the three common PAs in plants are putrescine (Put), spermidine (Spd), and spermine (Spm), with some plants also having thermo-spermine. Put is covalently conjugated (CC) to one or two aminopropyl groups to transform into Spd or Spm, respectively. Many processes and factors are involved in the biosynthesis of Spd or Spm and *S*-adenosylmethionine decarboxylase (SAMDC) is the most important one (Tiburcio et al., 1993). The research of Slocum (1991) showed that SAMDC was inhibited potently and exclusively by methylglyoxal-bis (guanylhydrazone) (MGBG). PAs control a variety of vital functions in plants, including growth and development (Guo et al., 2018; Yang et al., 2020). Over the last two decades, a bulk of data was accumulated providing explicit evidence that PAs play an essential function in tree growing and development, such as peach fruit development (Liu et al., 2006), seed development (Oliveira et al., 2016), and embryogenic culture (Jo et al., 2013) of Brazilian pine (*Araucaria angustifolia* B.), somatic embryogenesis of Scots pine (*Pinus sylvestris* L.) and Norway spruce (*Picea abies* L.) (Gemperlova et al., 2009; Salo et al., 2016), and aging in pine (*Pinus radiata* D.) and peach (*Prunus persica* L.) (Fraga et al., 2004). Furthermore, PAs play important functions in the tolerance of plants to environmental stresses (Shi et al., 2010; Grzesiak et al., 2013; Zheng et al., 2016; Du et al., 2018; Xu et al., 2018; Sun et al., 2021).

Polyamines (PAs) at physiological pH are polycations, bearing from 2 to 4 positive charges. By hydrogen bond and ionic bond, they can non-covalently be conjugated to bio-membrane phospholipids and acidic proteins (Sood and Nagar, 2003) and transformed into non-covalently conjugated PAs (non-CC PAs), which function in maintaining the integrity of bio-membrane and plasma membrane protein under environmental stress (Galston and Kaur-Sawhney, 1995). Furthermore, PA can be CC to glutamine residues of proteins to be transformed into CC PAs by the catalyzing of transglutaminase (TGase), which is strongly inhibited by o-phenanthroline. CC PAs function in modification of protein post-translating (Del Duca et al., 1995).

Polyamines (PAs) are the only organic polycations that are present in sufficient quantities under stress to play the role of channels blockers without compromising cell metabolism (Alcázar et al., 2010). At the same time, PAs could act as cofactors in the activation of H^+^-ATPase. Among immediate molecular targets for PAs, H^+^-ATPase is receiving growing attention (Zepeda-Jazo et al., 2011). Although existing data on the immediate effects of PAs on the H^+^-ATPase pumping activity are controversial (Pottosin and Shabala, 2014), the related researches are increasingly interesting. Free and conjugated PAs can modify (either activate or inhibit) the activity of the plasma membrane H^+^-ATPase. This may be due to the changes in the protein expression, membrane composition/stability, and redox state (Pottosin et al., 2021). There is an autoinhibitory domain in the H^+^-ATPase and the general mechanism of the H^+^-ATPase activation involves Thr-948 phosphorylation, which promotes 14-3-3 protein binding and relief of the autoinhibition (Svennelid et al., 1999; Jelich et al., 2001; Falhof et al., 2016). Furthermore, Athwal and Huber (2002) report that 14-3-3 protein can be activated by PAs because PAs (carrying positive charges) can be non-CC to the loop 8 (carrying negative charges) of 14-3-3 protein. Therefore, based on the previous work, one pathway mode of PAs regulating H^+^-ATPase might be put forward: PAs were conjugated to the loop 8 of 14-3-3 protein to activate the protein, and then the activated 14-3-3 protein could bind to the auto-inhibitory domain of H^+^-ATPase to activate H^+^-ATPase. On the other hand, PAs might be CC to the glutamine residues of H^+^-ATPase to maintain the conformation of the enzyme under stress. However, so far, the correlation between the activity of H^+^-ATPase and changes in non-CC polyamines and CC polyamines in the plasma membrane of the plum seedling roots under osmotic stress remains to be explored.

The present research aimed to elucidate the function of the conjugated PAs in the plasma membrane of plum seedling roots under PEG osmotic stress, which was used for simulating natural drought. Two plum (*Prunus salicina* L.) cultivars, which were different in drought tolerance, were used as experimental materials. The research mainly included the following items: the changes in free PA contents in roots, non-CC PAs and CC PAs in the plasma membrane, and H^+^-ATPase activity. Altogether, the results shown here should suggest that the conjugated PAs could function in stabilizing plasma membrane structure and therefore elevating H^+^-ATPase activity. To verify the hypothesis further, exogenous Spd, Spm, and two inhibitors (affecting the conjugated PA levels in the plasma membrane) were additionally used in the study.

## Materials and Methods

### Material Cultivation and Treatments

The experiment used two plum (*P. salicina* L.) cultivars (Ganli No. 5 and Suli No. 3) as materials. According to our preliminary experiments, Ganli No. 5, which grows mainly in the drought ecotope of North China, is drought-tolerant and Suli No. 3, which grows mainly in the rainy ecotope of South China, is drought-sensitive. The plum seeds were sterilized with 2% sodium hypochlorite for 30 min, then rinsed with distilled water, and germinated in black plastic pots (six seeds/pot) (bottom diameter/rim diameter/height: 20: 25: 35 cm) with small holes on the bottom, which contained quartz sand. The pots were put into a big plastic turnover box with half-strength Hoagland solution, which was replaced every 2 days with the same fresh solution. The big turnover box was placed into an environmental chamber with 26/16°C (day/night) temperature, 75% air humidity, and the cool-white, fluorescent lamps at 350 μmol m^–2^ s^–1^ quantum flux density for supplying 16 h photoperiod.

When the third pair of euphyllas began to appear, the full-strength Hoagland solution was applied to the seedling roots. The plum seedlings were uniformly thinned to three plants/pot, cultured for another 5 days, and then treated as follows.

Control: roots were grown in Hoagland solution with −0.15 MPa water potential for normal growth.
Treatment with PEG: roots were grown in Hoagland solution with PEG (−0.85 MPa).
Treatment with PEG + Spd: roots were grown in Hoagland solution with PEG (−0.85 MPa) and Spd (1 mM).
Treatment with PEG + Spm: roots were grown in Hoagland solution with PEG (−0.85 MPa) and Spm (1 mM).
Treatment with PEG + MGBG: roots were grown in Hoagland solution with PEG (−0.85 MPa) and MGBG (0.5 mM).
Treatment with PEG + o-phenanthroline: roots were grown in Hoagland solution with PEG (−0.85 MPa) and o-phenanthroline (0.2 mM).

**FIGURE 1:**
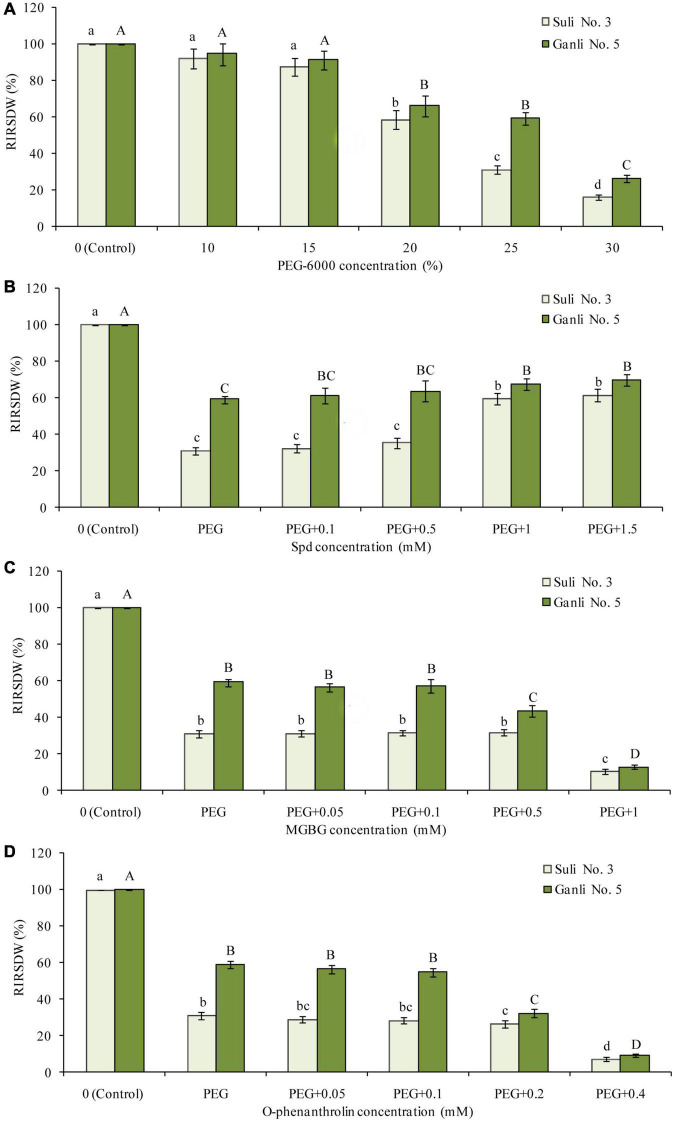
Effects of PEG **(A)**, PEG + Spd **(B)**, PEG + MGBG **(C)**, and PEG + o-phenanthroline **(D)** at the different concentrations on RIRSDW. The roots of plum seedlings were grown in Hoagland solutions with PEG-6000, Spd, MGBG, or o-phenanthroline at different concentrations. The data here were means (*n* = 9) ± standard error of 3 independent experiments. Significant differences between treatments are indicated with letters, with upper and lower letters for each cultivar (*P* < 0.05).

Polyethylene glycol (PEG)-6000 was used to decrease water potential, which was determined with an osmometer, Water Potential Instrument (Beijing Zhonghui Tiancheng Technology Co., LTD, Model: TEN60, Beijing, China). The results of dose determination of the reagents (PEG, Spd, Spm, MGBG, and o-phenanthroline) used in our research are displayed in Figure 1. Spd, Spm, MGBG, and o-phenanthroline were from Sigma Chemical Co. (St. Louis, MO, United States). All the Hoagland solutions mentioned above were renewed every day during the treatment period. After the seedlings were treated for 7 days, the plum roots were carefully rinsed thoroughly with deionized water to eliminate the residuary solution in the free spaces and on the surface of the plum seedling roots. The seedlings were drained with filter paper. The seedlings were sampled for assessment of relative increase rate of seedling dry weight (RIRSDW) and the same treated roots (7-mm-long apices of white roots) from the seedlings un-sampled for RIRSDW were sampled for the other indexes.

### Assessment of Relative Increase Rate of Seedling Dry Weight

To assess the effects of all treatments on the growth of plum seedlings, we used RIRSDW as a different growth indicator. RIRSDW was calculated according to the method of Liu et al. (2004):

Increase rate of seedling dry weight (IRSDW) = (W_7_ − W_0_) / W_0_ (in the formula, W_7_ and W_0_ mean the dry weight of seedlings treated for 7 and 0 days, respectively). To counteract the diversity between the two cultivars, the RIRSDW of every plum cv. was calculated by the following formula:


RIRSDW(%)=(IRSDWoftreatmentofeverycv./IRSDWofcontrolofthesamecv.)×100.

### Assessment of Relative Water Content of Root

The relative water content of root (RWCR) was calculated by the following formula:

RWCR (%) = (fresh weight − dry weight) / (saturation weight − dry weight) × 100 (in the formula, fresh weight, saturation weight, and dry weight represent the weight of the plum seedling roots treated for 7 days, respectively). After being weighed for fresh weight, the fresh roots were immersed in distilled water immediately for fully absorbing water until the root weight was constant. Then, the weight was defined as saturation weight. The samples of fresh roots were put into an oven, killed out at 110°C for 20 min, and then baked at 65°C until the root weight was constant. The constant root weight was regarded as dry weight.

### Determination of Relative Plasma Membrane Permeability

Plasma membrane injury is performed by relative plasma membrane permeability (RPMP). Plum seedling root RPMP was assessed following the method of Jahan et al. (2019) with proper adjustments. The plum seedling roots (0.2 g) were put into a test tube with 10 ml of de-ionized water. Then, in a shaker under dark conditions, the tube was incubated at 25°C in water for 3 h. With a portable Conductivity Meter (Shanghai Thunder Magnetic Scientific Instrument Co., LTD, Model: DDBJ-350, Shanghai, China), initial electrical conductivity (IEC) of the sample was detected. Subsequently, the sample was boiled for 15 min at 100°C to release all electrolytes, and then cooled to 25°C. The final electrical conductivity of the medium was measured and marked as FEC. Meanwhile, the conductivity of the de-ionized water was determined and marked as DEC. RPMP was determined by the following formula:


RPMP(%)=(IEC-DEC)/(FEC-DEC)×100.

### Determination of Malondialdehyde

Lipid peroxidation in root cells is displayed by the content of malondialdehyde (MDA). In the study, MDA content was detected by the method of Alexieva et al. (2001) with minor modifications. With 1.5 ml trichloroacetic acid solution (w/v: 0.1%), 0.2 g root samples were ground. Then, the homogenate was centrifuged at 13,000 × *g* for 25 min at 4°C. The 1 ml supernatant was added to an aliquot 1 ml trichloroacetic acid with thiobarbituric acid (w/v:0.65%). The mix was boiled for 20 min at 95°C, and it was kept cooling down on fragmented ice. Subsequently, the sample was centrifuged for 20 min at 4,000 × *g*. At 532 and 600 nm, the MDA level was determined with a spectrophotometer (Zhongke Ruijie Technology Co., LTD, Beijing, China, Model: RV-1100).

### Purification of Plasma Membrane Vesicles and Assessing of H^+^-ATPase Activity

Plasma membrane vesicles of plum seedling roots were isolated by density gradient centrifugation using the method of Qiu and Su (1998) with some adjustments. In brief, 2 g seedling root was homogenized in prepared solution, which was composed of sorbitol (0.2 M), KCl (0.1 M), ethylene glycol tetraacetic acid (EGTA) (4 mM), dithiothreitol (DTT) (2 mM), phenylmethanesulfonyl fluoride (PMSF) (1 mM), glycerol (20%, v/v), polyvinyl pyrrolidone (PVP) (1.5%, w/v), bovine serum albumin (BSA) (0.3%, w/v), K_2_S_2_O_5_ (0.2 M) and Tris-hydroxyethyl piperazine ethyl sulfonic acid (Hepes) (60 mM, pH 7.8). The sample was filtered with four layers of gauze. The filtrate was centrifugated at 12,000 × *g* for 20 min. To get plasma membrane microsomal precipitate, the supernatant was centrifugated again for 35 min at 80,000 × *g*. The microsomal precipitate was gently resuspended in a buffer, which was composed of sucrose (0.2 M), DTT (2 mM), EGTA (2 mM), KCl (15 mM), BSA (w/v:0.2%), and Tris-Hepes (2.5 mM, pH 7.5). The suspending sample was further purified according to Qiu and Su (1998). According to Widell and Larsson’s (1990) method, the plasma membrane vesicle purity was assessed. In the study, the purity of the isolated plasma membrane was reliable. The H^+^-ATPase activity was assessed by detecting inorganic phosphate content from ATP using the method of Ohinishi et al. (1975) and Qiu and Su (1998) with some adjustments. The assaying reaction solution was composed of KNO_3_ (60 mM), Hepes (30 mM), ATP-Na_2_ (4 mM), MgSO_4_ (2.5 mM), NaN_3_ (1.5 mM), Tris-Na_2_MoO_4_ (0.2 mM, pH 6.6) and 7 μg proteins of plasma membrane vesicles. The sample was incubated for 35 min at 37°C and the reaction was quenched by trichloroacetic acid (15%, w/v).

### Determination of Free Polyamines in the Roots of Plum Seedlings

Free PA was determined by the method of Sharma and Rajam (1995) with proper adjustments. One gram seedling roots were ground in 4 ml perchloric acid water solution (5%, *v*/*v*) and in a 5°C refrigerator the homogenate was incubated for 1 h. The sample was centrifuged at 25,000 × *g* for 35 min. With the collected supernatant, the levels of free PAs were assessed. With benzoyl chloride, the supernatant was derivatized according to the Di Tomaso et al. (1989) method. Free PA was detected by high-performance liquid chromatography (HPLC), using a column of reverse-phase (C-18) as separation column and 254 nm as detecting wavelength (Waters 2695, United States).

### Determination of Non-covalently Conjugated Polyamine Levels

Non-covalently conjugated PAs in the plasma membrane was quantified by the method of Sharma and Rajam (1995) with proper modifications. The membrane vesicles purified as described above were added with 3 ml perchloric acid (w/v: 5%) and the samples were centrifugated for 40 min at 28,000 × *g*. Non-CC PAs were in the supernatant. With benzoyl chloride, the non-CC PAs were derivatized and quantified by HPLC.

### Determination of Plasma Membrane Protein and Covalently Conjugated Polyamine Levels

To isolate the protein in the plasma membrane, stock triton X-100 solution (10%, v/v) was dropwise added into the part of the prepared plasma membrane vesicle until the final concentration of triton lowered to 1%. With an ultrasonic disintegrator (Hangzhou Farant Ultrasonic Technology Co., LTD, Hangzhou, China, Model: 200-W), the sample was ultra-sonicated to isolate protein in the plasma membrane. Afterward, it was centrifugated at 4°C for 40 min at 25,000 × *g*. Just in the supernatant, the soluble plasma membrane protein was detected by the method of Bradford (1976), with BSA being used as standard.

Covalently conjugated PAs in the plasma membrane was quantified by the method of Sharma and Rajam (1995) with proper modifications. The membrane protein extract mentioned above was mixed with perchloric acid dropwise until the terminal concentration of perchloric acid reached 5%. Afterward, the mix was centrifugated for 50 min at 30,000 × *g*. The supernatant was removed and perchloric acid (w/v: 5%) was added to the precipitate to make it re-suspended. Then the solution was transferred into an ampoule and an equal volume of 12 N hydrogen chloride (HCl) was added to the solution. The ampoule was put in a drying oven after it had been sealed. The sample was hydrolyzed for 24 h at 110°C, filtrated, and dried with warm air at 70°C. The pellet with CC PAs was re-dissolved with perchloric acid (w/v: 5%). After that, CC PAs in the solution were derivatized with benzoyl chloride and quantified by HPLC.

### Detection of *S*-Adenosylmethionine Decarboxylase Activity in Plum Seedling Root

*S*-adenosylmethionine decarboxylase activity was detected by the method of Kaur-Sawhney and Shin (1982) with some adjustments. The roots from random plum seedling samples were homogenized in two volumes of 100 mM phosphate buffer at pH 7.6. The homogenates were centrifuged at 26,000 × *g* for 15 min at 4°C. The resulting clear supernatant fraction was used as SAMDC extract. The assay mix was composed of Tris–HCl buffer (0.1 M, pH 8.3), EDTA (0.2 mM), 2-mercaptoethanol (2 mM), and the seedling root enzyme extracting solution. With a glass scintillation vial, the reaction was carried out to gather the released ^14^CO_2_. The assay mix was added into using *S*-adenosyl-L-[carboxyl-^14^C] methionine (0.5 nmol) after the reaction went on at 30°C firstly for 5 min. After the assay mix in the capped vial reacted again for 25 min, the reaction was quenched with 0.4 ml KH_2_PO_4_ (1 M). Again, the vial was closed at once, and at 37°C, it was shaken for 1 h. ^14^CO_2_ was collected into a vial and determined. The amount of the formation of 1 μl ^14^CO_2_ min^–1^ catalyzed by SAMDC enzyme was defined as one enzyme activity unit. The enzyme activity of the control was assessed with boiled root extracts.

### Transglutaminase Activity Assessing in Plum Seedling Root

Transglutaminase activity was detected by the method of Icekson and Apelbaum (1987) with proper modifications. The plum seedling root was homogenized by using four volumes of Tris–HCl (100 mM, pH 8.8) at 4°C. Then the homogenate was centrifuged for 20 min at 1,500 × *g* under 4°C condition. TGase activity was assessed in the supernatant. The rate of incorporation of ^3^[H] Put into precipitated proteins was detected to determine the TGase activity. Finally, 1 nmol of ^3^[H] Put mg^–1^ protein h^–1^ was defined as one activity unit of TGase enzyme.

### Statistical Analysis

Three independent experiments were performed and, in each of them, three samples were taken for data collecting. Therefore, the data in the article were means (*n* = 9) ± standard error of 3 independent experiments. The software of SPSS 16 (SPSS Inc., Armonk, NY, United States) and Microsoft Excel software was used to analyze the data. The significant changes between means among treatments were assessed by two-way ANOVA and Dunnett’s multiple range tests (*P* < 0.05).

## Results

### Effects of Polyethylene Glycol, Polyethylene Glycol + Spermidine, Polyethylene Glycol + Spermine, Polyethylene Glycol + Methylglyoxal-bis Guanylhydrazone, and Polyethylene Glycol + O-Phenanthroline at the Different Concentrations on Relative Increase Rate of Seedling Dry Weight

The obvious reaction of plants to environmental stresses is shown as growth-inhibiting and the plant drought resistance is closely related to growth, which is determined by the accumulation of dry matter (Kaur and Asthir, 2017). Therefore, the index RIRSDW was used to estimate the tolerance of plum seedlings to osmotic stress and the effectiveness of the treatment with every reagent at the different concentrations used in this research (Figure 1). After the plum seedlings were treated with 25% PEG (−0.85 MPa) for 7 days, there was a marked difference between the two plum cultivars in the index (Figure 1A). RIRSDW of Suli 3 was reduced by 69.1%, compared with the control under the normal growth conditions, whereas RIRSDW of Ganli 5 was reduced by 40.5%. Therefore, it could be preliminarily inferred that plum Ganli 5 was drought tolerant and Suli 3 was drought sensitive. The inference could be subsequently verified further with the other indexes, RWCR, RPMP, and MDA content. Indeed, −0.85 MPa osmotic potential was applied in the research. Furthermore, under osmotic stress, treatments with exogenous Spd at 1 mM concentration could markedly enhance the tolerance of plum seedlings to osmotic stress, especially the tolerance of drought-sensitive Suli No. 3 (Figure 1B). Treatment effects below this dose were not statistically significant. Therefore, exogenous Spd at 1 mM concentration was adopted in the research. The effect of Spm at the different concentrations on RIRSDW was the same as that of Spd (data not shown). Similarly, treatments with MGBG at 0.5 mM (Figure 1C) or o-phenanthroline at 0.2 mM concentration (Figure 1D) could decrease markedly the tolerance of plum seedlings to osmotic stress, especially the tolerance of drought-tolerant Ganli No. 5. Treatment effects below this dose were not statistically significant and treatment effects above this dose might lead to the seedlings stopping growing. Therefore, 0.5 mM MGBG and 0.2 mM o-phenanthroline were used in the research.

### Changes in Relative Increase Rate of Seedling Dry Weight and Relative Water Content of Root

**FIGURE 2:**
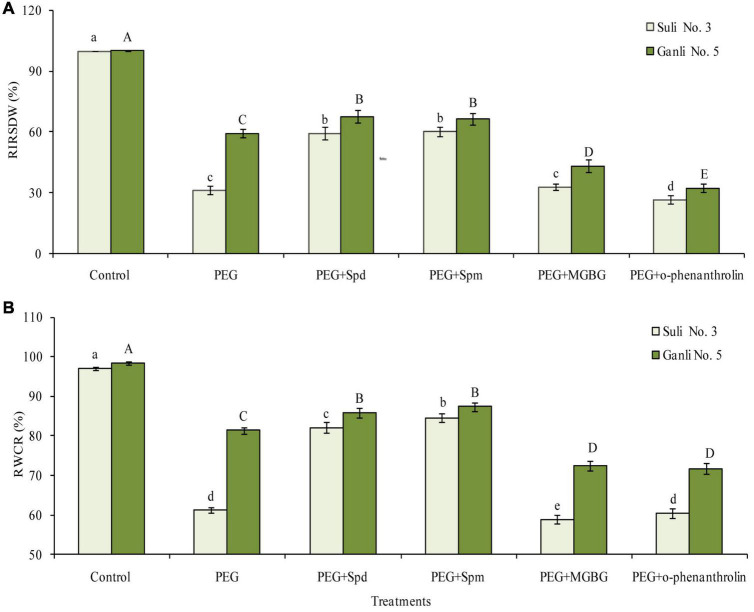
Changes in RIRSDW **(A)** and RWCR **(B)** of plum seedlings under PEG, exogenous Spd, Spm, MGBG, and o-phenanthroline. Control: treatment without PEG or the other reagents (–0.15 MPa); PEG: treatment with PEG (–0.85 MPa); PEG + Spd: treatment with PEG and Spd (1 mM); PEG + Spm: treatment with PEG and Spm (1 mM); PEG + MGBG: treatment with PEG and MGBG (0.5 mM); PEG + o-phenanthroline: treatment with PEG and o-phenanthroline (0.2 mM). The data here were means (*n* = 9) ± standard error of 3 independent experiments. Significant differences between treatments are indicated with letters, with upper and lower letters for each cultivar (*P* < 0.05).

Under osmotic stress, RIRSDW (Figure 2A) and RWCR (Figure 2B) of Suli 3 (drought-sensitive) and Ganli 5 (drought-resistant) decreased. The changes in Suli 3 cv. were more severe than that in Ganli 5 cv. RIRSDW and RWCR of Suli 3 were reduced by 69.1 and 37.1%, respectively, compared with the control under the normal growth conditions, whereas RIRSDW and RWCR of Ganli 5 were reduced by 40.5 and 17.5%, respectively. Under osmotic stress, the decreases of RIRSDW and RWCR of Suli 3 cv. were significantly inhibited by exogenous Spd treatment. Effects of exogenous Spd on the two indexes of Ganli 5 cv. were mild. Exogenous Spm treatment had the same effects on the two cultivars under osmotic stress as exogenous Spd. Conversely, the treatment with MGBG or o-phenanthroline, which inhibited PA biosynthesis, aggravated the decreases of RIRSDW and RWCR of the two cultivars under osmotic stress. The effect of MGBG or o-phenanthroline on Suli 3 was slight.

### Changes in Relative Plasma Membrane Permeability and Malondialdehyde

**FIGURE 3:**
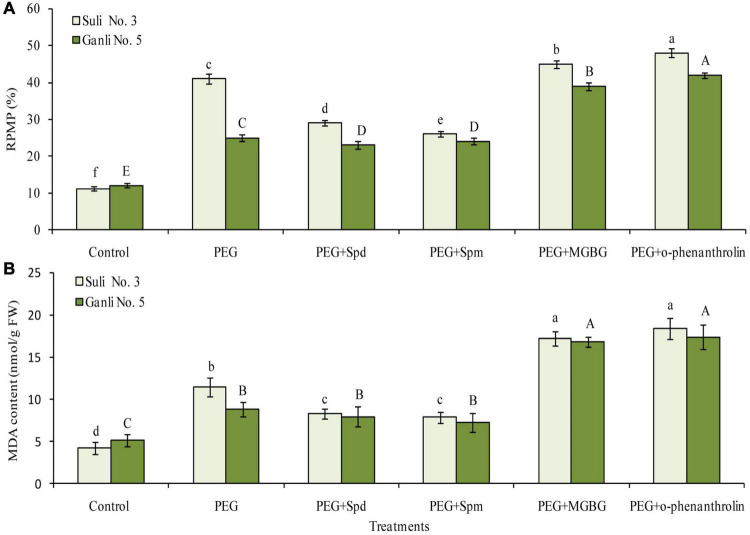
Changes in RPMP **(A)** and the content of MDA **(B)** of plum seedling roots under PEG, exogenous Spd, Spm, MGBG, and o-phenanthroline. Control: treatment without PEG or the other reagents (–0.15 MPa); PEG: treatment with PEG (–0.85 MPa); PEG + Spd: treatment with PEG and Spd (1 mM); PEG + Spm: treatment with PEG and Spm (1 mM); PEG + MGBG: treatment with PEG and MGBG (0.5 mM); PEG + o-phenanthroline: treatment with PEG and o-phenanthroline (0.2 mM). The data here were means (*n* = 9) ± standard error of 3 independent experiments. Significant differences between treatments are indicated with letters, with upper and lower letters for each cultivar (*P* < 0.05).

To estimate the damage degree of osmotic stress to the plasma membrane, RPMP and the content of MDA were used in the study. From Figure 3A, it was observed that the RPMP of the two cultivars increased sharply under osmotic stress. The RPMP of Suli 3 and Ganli 5 increased by around 2.6 and 1.2 times, respectively, indicating that osmotic stress destroyed the plasma membrane integrity of Suli 3 more severely than that of Ganli 5. Application of Spd or Spm was effective for overcoming harsh impacts of osmotic stress on the plasma membrane integrity, as shown by a substantial reduction of RPMP, compared with untreated plants. MGBG treatment combined with osmotic stress further brought about the RPMP increases in Suli 3 and Ganli 5 of around 3.2 and 2.5 times, respectively, with respect to the control. Under osmotic stress, the results of o-phenanthroline treatment were the same as those of MGBG treatment. O-phenanthroline treatment combined with osmotic stress further brought about RPMP increases in Suli 3 and Ganli 5 of around 3.5 and 2.4 times, respectively, compared with the control. The effects of osmotic stress, exogenous Spd, Spm, MGBG, and o-phenanthroline on the level of MDA (Figure 3B) were in accordance with those on RPMP, showing that the change in membrane lipid peroxidation level was consistent with the change in plasma membrane permeability.

### Changes in the Levels of Free Polyamine in Plum Roots

**FIGURE 4:**
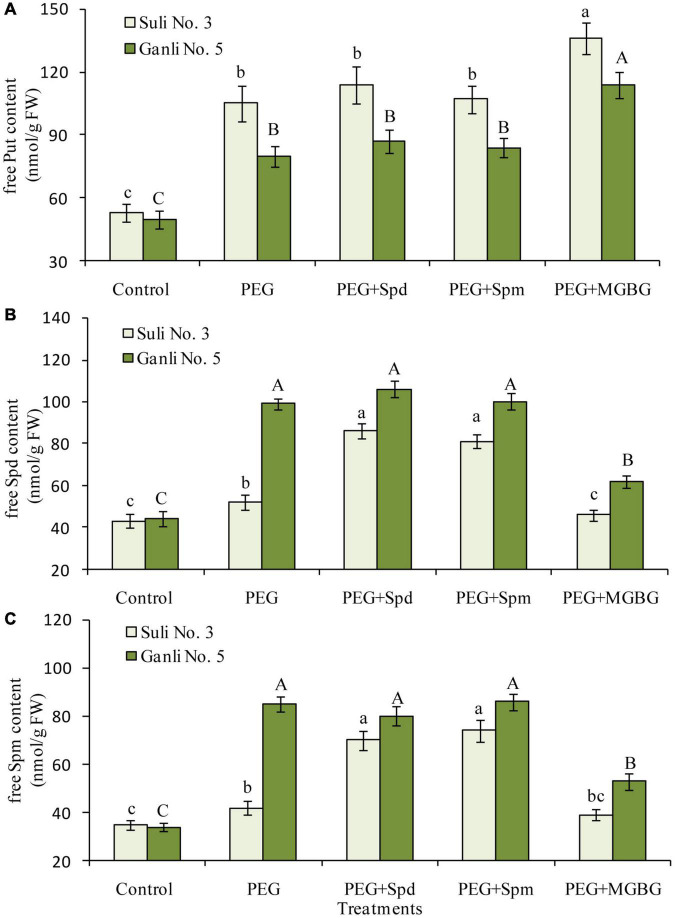
Changes in the contents of free Put **(A)**, free Spd **(B)**, and free Spm **(C)** in the plum seedling roots under PEG, exogenous Spd, Spm, and MGBG. Control: treatment without PEG or the other reagents (–0.15 MPa); PEG: treatment with PEG (–0.85 MPa); PEG + Spd: treatment with PEG and Spd (1 mM); PEG + Spm: treatment with PEG and Spm (1 mM); PEG + MGBG: treatment with PEG and MGBG (0.5 mM). The data here were means (*n* = 9) ± standard error of 3 independent experiments. Significant differences between treatments are indicated with letters, with upper and lower letters for each cultivar (*P* < 0.05).

There mainly were three free polyamines (Put, Spd, and Spm) in plum seedling roots. Effects of osmotic stress on free PA levels in the roots differed notably between the two plum cv. (Figure 4). Free Put in the roots of osmotic stress-treated Suli 3 was 1.9 times higher than that in the control, and in osmotic stress-treated Ganli 5, it was 1.6 times higher than that in the control (Figure 4A). At the same time, free Spd (Figure 4B) and Spm (Figure 4C) levels increased markedly by 125 and 150%, respectively, in the roots of the drought-tolerant Ganli 5, whereas in the drought-sensitive Suli 3, they only increased by 21 and 20%, respectively. The results indicated that free Spd and free Spm might be involved in plum seedling tolerance to osmotic stress. If this was the case, the treatment of plum seedlings with an inhibitor of Spd and Spm biosynthesis might weaken the tolerance to osmotic stress. To confirm this hypothesis, we used MGBG, an inhibitor of SAMDC. The result of PA analysis indicated that the increases in free Spd and Spm levels induced by osmotic stress were inhibited (Figures 4B,C) and the stress-induced damage of plum seedlings was aggravated by the treatment with MGBG, as judged by the decreased RIRSDW (Figure 2A) and RWCR (Figure 2B), and increased RPMP (Figure 3A) and MDA (Figure 3B) content. Furthermore, under osmotic stress, exogenous Spd and Spm were used in the research to verify the hypothesis. The treatment of plum seedlings with exogenous Spd or Spm led to further marked increases in the contents of free Spd and free Spm in roots (Figures 4B,C), and simultaneously, resulted in alleviating greatly the damage to seedlings induced by osmotic stress, as judged by the much-increased RIRSDW (Figure 2A) and RWCR (Figure 2B), and decreased RPMP (Figure 3A) and MDA (Figure 3B) content.

### Changes in Non-covalently Conjugated -Polyamine Contents in the Plasma Membrane of Plum Seedling Roots

**FIGURE 5:**
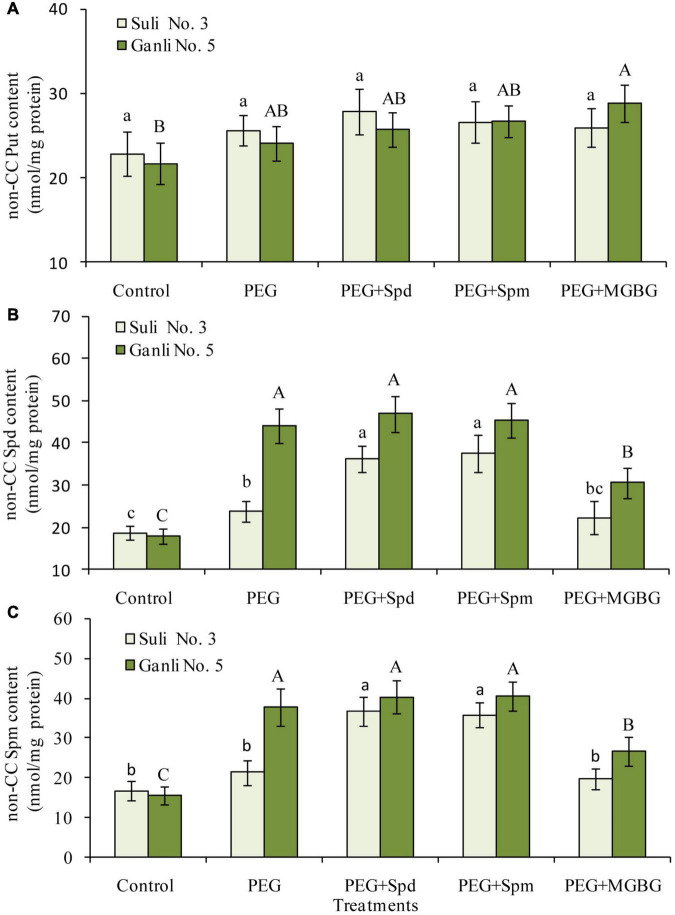
Changes in the contents of non-CC Put **(A)**, non-CC Spd **(B)**, and non-CC Spd **(C)** in plasma membrane from plum seedling roots under PEG, exogenous Spd, Spm, and MGBG. Control: treatment without PEG or the other reagents (–0.15 MPa); PEG: treatment with PEG (–0.85 MPa); PEG + Spd: treatment with PEG and Spd (1 mM); PEG + Spm: treatment with PEG and Spm (1 mM); PEG + MGBG: treatment with PEG and MGBG (0.5 mM). The data here were means (*n* = 9) ± standard error of 3 independent experiments. Significant differences between treatments are indicated with letters, with upper and lower letters for each cultivar (*P* < 0.05).

From Figure 4, free Spd and Spm in the plum seedling roots functioned in enhancing plum seedlings’ tolerance against osmotic stress. At physiological pH, PAs might locate in the cell compartments in the form of conjugated PAs due to their positive charges. Therefore, in the present research, it was necessary to investigate the contents of non-CC Spd and non-CC Spm in the plasma membrane of the plum seedling roots to further elucidate their function in enhancing the tolerance of plum seedlings to osmotic stress. Osmotic stress treatment led to the increases in the levels of non-CC Spd (Figure 5B) and non-CC Spm (Figure 5C) in the plasma membrane of both plum cultivar roots, and the contents of non-CC Spd and non-CC Spm of Ganli 5 (drought tolerant) rose more significantly than those of Suli 3 (drought-sensitive). The level of non-CC Put in Suli 3 rose higher than that in Ganli 5 (Figure 5A). Exogenous Spd or Spm treatment induced significant increases of the levels of non-CC Spd and non-CC Spm in plasma membrane from Suli 3 roots under osmotic stress. However, the increases were negligible in Ganli 5. Treatment with MGBG significantly inhibited the increases in the levels of non-CC Spd and non-CC Spm induced by osmotic stress in the plasma membrane of the plum roots of Ganli 5 and the effect of MGBG on Suli 3 was mild (Figures 5B,C). Under osmotic stress, the treatment with exogenous Spd, Spm, or MGBG affected the level of non-CC Put slightly in two plum cultivars.

### Changes in Covalently Conjugated Polyamines in the Plum Root Plasma Membrane

**FIGURE 6:**
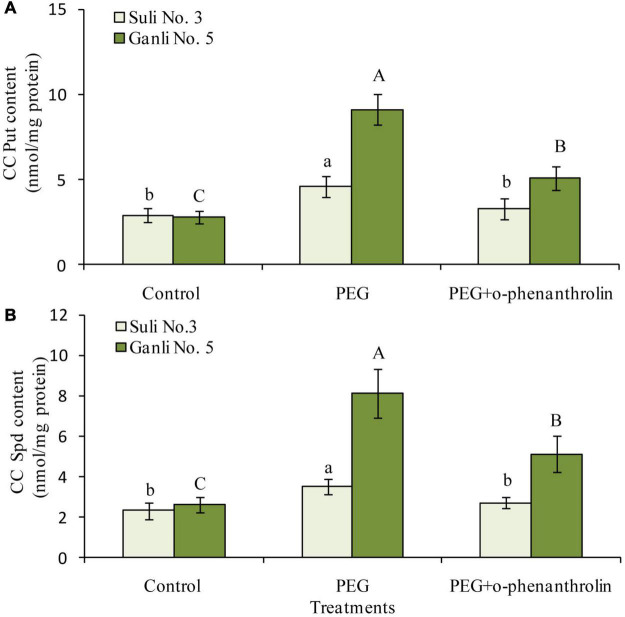
Changes in the contents of CC Put **(A)** and CC Spd **(B)** in the plasma membrane of plum seedling roots under PEG and o-phenanthroline. Control: treatment without PEG or the other reagents (–0.15 MPa); PEG: treatment with PEG (–0.85 MPa); PEG + o-phenanthroline: treatment with PEG and o-phenanthroline (0.2 mM). The data here were means (*n* = 9) ± standard error of 3 independent experiments. Significant differences between treatments are indicated with letters, with upper and lower letters for each cultivar (*P* < 0.05).

With regards to CC PAs, under osmotic stress, the contents of CC Put and CC Spd in the plasma membrane of the plum seedling roots of both cultivars could be detected (Figure 6). However, the level of CC Spm could not be detected because it might be too low. Osmotic stress significantly induced the increases in CC Put (Figure 6A) and CC Spd (Figure 6B) contents of Ganli 5 by 219 and 230%, respectively, but the treatment only had a slight effect on Suli 3, elevating the contents of CC Put and CC Spd by 59 and 49%, respectively. The increases of CC Put (Figure 6A) and CC Spd (Figure 6B) levels induced by osmotic stress in the plasma membrane of both plum cultivars were inhibited by o-phenanthroline treatment and this effect was greater in Ganli 5 than in Suli 3 when both cultivars were subjected to osmotic stress (Figure 6).

### Change in *S*-Adenosylmethionine Decarboxylase Activity in Plum Seedling Roots

**FIGURE 7:**
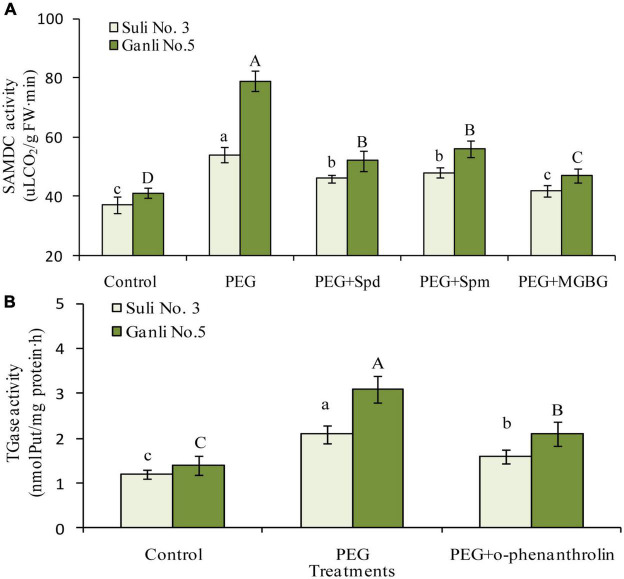
Changes in SAMDC **(A)** and TGase **(B)** activities. Control: treatment without PEG or the other reagents (–0.15 MPa); PEG: treatment with PEG (–0.85 MPa); PEG + Spd: treatment with PEG and Spd (1 mM); PEG + Spm: treatment with PEG and Spm (1 mM); PEG + MGBG: treatment with PEG and MGBG (0.5 mM); PEG + o-phenanthroline: treatment with PEG and o-phenanthroline (0.2 mM). The data here were means (*n* = 9) ± standard error of 3 independent experiments. Significant differences between treatments are indicated with letters, with upper and lower letters for each cultivar (*P* < 0.05).

*S*-adenosylmethionine decarboxylase is the key enzyme of Spd or Spm biosynthesis. Therefore, to further elucidate the changes in Spd and Spm in the plum seedling roots, the change in SAMDC activity was assayed in the research (Figure 7A). Under osmotic stress, the SAMDC activity increased by 92.7 and 46.1% in the roots of drought-tolerant Ganli 5 and drought-sensitive Suli 3, respectively, implying that SAMDC activity was related to the tolerance. Osmotic-induced increase of SAMDC activity was inhibited by the treatment with exogenous Spd or Spm, due to the feedback inhibition of the elevated products of the enzyme. Under osmotic stress, MGBG treatment inhibited SAMDC activity in the roots of Ganli 5 and Suli 3 by 42.0 and 25.8%, respectively, in contrast with the samples subjected only to osmotic stress (Figure 7A).

### Change in the Transglutaminase Activity of Plum Seedling Roots

Change in the activity of TGase, which catalyzes CC-PA biosynthesis, was shown in Figure 7B. Under osmotic stress, the TGase activity in the roots of Ganli 5 and Suli 3 increased by 126 and 76%, respectively, suggesting that TGase activity was related to tolerance. The osmotic stress-induced increase of TGase activity in plum seedling roots was inhibited by o-phenanthroline treatment and the activity of Ganli 5 and Suli 3 was inhibited by 35 and 26%, respectively (Figure 7B).

### Change in H^+^-ATPase Activity in the Root Plasma Membrane of Plum Seedlings

**FIGURE 8:**
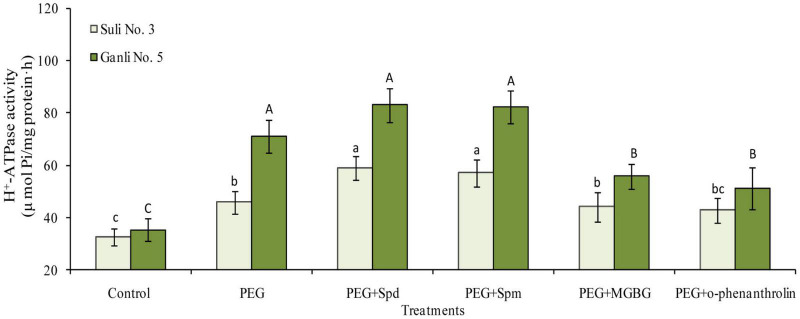
Changes in the activity of root plasma membrane H^+^-ATPase of plum seedlings under PEG, exogenous Spd, Spm, MGBG, and o-phenanthroline. Control: treatment without PEG or the other reagents (–0.15 MPa); PEG: treatment with PEG (–0.85 MPa); PEG + Spd: treatment with PEG and Spd (1 mM); PEG + Spm: treatment with PEG and Spm (1 mM); PEG + MGBG: treatment with PEG and MGBG (0.5 mM); PEG + o-phenanthroline: treatment with PEG and o-phenanthroline (0.2 mM). The data here were means (*n* = 9) ± standard error of 3 independent experiments. Significant differences between treatments are indicated with letters, with upper and lower letters for each cultivar (*P* < 0.05).

Under osmotic stress, the H^+^-ATPase activity in the plasma membrane of plum seedling roots of drought-tolerant Ganli 5 and drought-sensitive Suli 3 increased by 106 and 39%, respectively, showing that the osmotic stress-induced increase in the former was much more marked than that in the latter (Figure 8). The treatment with exogenous Spd significantly enhanced the osmotic stress-induced increase of plasma membrane H^+^-ATPase activity in roots of Suli 3 and Ganli 5 by 31 and 15%, respectively, indicating that the effect of exogenous Spd on the enzyme activity of Suli 3 was more marked than that of Ganli 5. The result of exogenous Spm application was similar to that of Spd (Figure 8). MGBG treatment markedly inhibited the stress-induced increase of the enzyme activity in the plasma membrane of Ganli 5 by 24%, and o-phenanthroline treatment inhibited it by 29%, while MGBG or o-phenanthroline treatment brought about a slight effect on the enzyme activity of Suli 3 (Figure 8).

## Discussion

### Verification of the Drought Tolerance of the Two Plum Cultivars

Global climate change is expected to increase the frequency and severity of drought events in many regions worldwide, severely affecting plant production. Plum trees are widely distributed throughout the world. Breeding and selecting the drought-resistance cultivar is one of the important measurements which are applied to plum planting in arid and semi-arid regions. In China, Suli 3 plum cv. is mainly planted in rainy ecotope of South and Central China, such as in Jiangsu, Anhui, Hunan, and Zhejiang province, whereas Ganli plum 5 cv. is mainly distributed in drought and semi-drought ecotopes of North China, such as in Henan, Hebei, Shanxi, and Gansu province. So, it could be anticipated that Suli 3 cv. was drought-sensitive and Ganli 5 cv. was drought-resistance. To verify the drought tolerance of the two plum cultivars, we examined the indexes of RIRSDW, RWCR, RPMP, and MDA content in the study, since the obvious reaction of plants to environmental stresses is shown as growth-inhibiting and the plant drought resistance is closely related with relative water content and growth, which is determined by the accumulation of biomass and dry matter (Kaur and Asthir, 2017). From Figure 2A, it could be shown that the osmotic stress-induced decrease of the RIRSDW (Figure 2A) of Suli 3 cv. was more marked than that of Ganli 5 cv. Therefore, it could be verified that plum Ganli 5 was drought tolerant and Suli 3 was drought sensitive. Furthermore, the differences in the tolerance of the two plum cultivars were further confirmed with the additional indexes of RWCR (Figure 2B), RPMP (Figure 3A), and MDA content (Figure 3B). Since the different sensitivity of the two plum cultivars had been verified, we could proceed to compare the levels of free and conjugated PAs in both cultivars in order to verify the possible involvement of PAs conjugated to the plasma membrane of root cells in the tolerance of the plum seedlings to osmotic stress.

### The Function of Free Polyamines in Plum Seedling Roots Under Osmotic Stress

Understanding the role of PAs in plant adaptive responses to drought might be instrumental in breeding crops with improved tolerance to drought stress. To elucidate the function of conjugate PAs, it is necessary to elaborate firstly on the roles which the free PAs in plum seedling roots played in the osmotic stress tolerance. From Figure 4, it could be concluded that free Spd and free Spm functioned in the resistance of plum seedlings to osmotic stress, and applications of exogenous Spd, Spm, and inhibitor MGBG provided further testimonies, with the activities of SAMDC, which is inhibited by MGBG potently and exclusively (Figure 7). Some previous studies have suggested that Put build-up could cause the injury induced by drought stress (Pedrol et al., 2000) and other abiotic stresses (Slocum, 1991; Rodriguez et al., 2001). Nevertheless, recent researches showed that the application of exogenous Put could enhance the tolerance of plants to drought stress (Ebeed et al., 2017; Hassan et al., 2020; Upadhyay et al., 2021) and chilling stress (Ahmad and Ali, 2019). Therefore, in the resistance of plants to environmental stresses, the precise significance of putrescine is still not clear. However, the functions of free spermidine and spermine in plants under drought stress have been well documented (Yamaguchi et al., 2007; Kubis, 2008). In addition, Farooq et al. (2009) reported that spermine played the most effective function in elevating drought resistance because, among the PAs, it carried the most positive charges. The researches on the mechanism underlying the function of free Spd and free Spm in osmotic stress tolerance are increasingly attractive. Since that PAs exist in all the compartments of the plant cells, the significance of the non-CC Spd and non-CC Spm conjugated to the plasma membrane was probed in the study.

### Significance of Non-covalently Conjugated Spermidine and Spermine in Plasma Membrane Under Osmotic Stress

The study of Gupta et al. (2013) reported that PAs might non-covalently be conjugated to cellular components to be transformed into non-CC PAs and function in the plant resistance to many stresses. In the research, from Figures 5B,C, it could be seen that the contents of non-CC Spd and non-CC Spm in Ganli 5 (drought tolerant) rose more markedly than those in Suli 3 (drought-sensitive) under osmotic stress. Conversely, non-CC Put level in drought-tolerant cv. did not increase as much as that in drought-sensitive cv. (Figure 5A). Therefore, it was inferred that non-CC Spd and non-CC Spm, rather than non-CC Put, would be associated with the resistance of plum seedlings to osmotic stress. The suggestion was further supported by the additional experiments with exogenous Spd, Spm, and MGBG (Figure 5). The treatment with exogenous Spd and Spm obviously increased not only the non-CC Spd and non-CC Spm levels in the plasma membrane of drought-sensitive Suli 3 cv. (Figure 5), but also the resistance of the plum cv., as judged by the indexes of RIRSDW (Figure 2A), RWCR (Figure 2B), RPMP (Figure 3A), and MDA content (Figure 3B). The treatment with MGBG inhibitor markedly decreased not only the levels of non-CC Spd and non-CC Spm of the plasma membrane of Ganli 5 cv. (Figure 5), but also the resistance of the plum cv. (Figures 2, 3). The finding was in accordance with the results of Dutra et al. (2013), showing that due to their more positive charges than Put, Spd and Spm could non-covalently be conjugated to the plasma membrane to be transformed into non-CC Spd and non-CC Spm more easily and maintain configuration and function of pro-embryogenic cells.

### Significance of Covalently Conjugated Polyamines in Plasma Membrane Under Osmotic Stress

Besides non-CC PAs in the plasma membrane, there exist CC PAs conjugated to the residues of the proteins in the plasma membrane. So, in the research, the function of CC PAs in the roots of plum seedlings was also elucidated. Under osmotic stress, the contents of CC Put (Figure 6A) and Spd (Figure 6B) in the plasma membrane of drought-tolerant Ganli 5 cv. rose more significantly. From the results, we could conclude that the two conjugated PAs might be related to the resistance of plum seedlings against osmotic stress. The inference was further confirmed by the additional experiment with o-phenanthroline, which potently inhibits the activity of TGase, a key enzyme of CC PA biosynthesis (Del Duca et al., 1995). O-phenanthroline treatment substantially decreased not only CC Put and Spd contents in the plasma membrane of Ganli 5 (Figure 6), but also the resistance of the plum cv., as judged by the indexes of RIRSDW (Figure 2A), RWCR (Figure 2B), RPMP (Figure 3A), and MDA content (Figure 3B). Our notion is supported by Del Duca et al. (1995), who reported that CC PAs functioned in modification of protein post-translating. Furthermore, the changes in the contents of CC Put and Spd in the plasma membrane were in accordance with the changes in the TGase activity in the roots under the combined treatments with osmotic stress and inhibitor o-phenanthroline (Figure 7B), which confirmed our finding additionally.

### Relationship Between Conjugated Polyamines and H^+^-ATPase in Plasma Membrane Under Osmotic Stress

Change in plasma membrane H^+^-ATPase activity under water stress remains controversial (Michelet et al., 1994; Qiu and Zhang, 2000). Michelet et al. (1994) indicate that the H^+^-ATPase activity in the plasma membrane is enhanced under drought stress, while Qiu and Zhang (2000) argued that the activity of H^+^-ATPase from soybean hypocotyls is inhibited under water stress. However, osmotic stress increased the enzyme activity in the roots of plum seedlings in the present research. In addition, the increase was more significant in drought-tolerant cv. than in drought-sensitive cv. (Figure 8), implying that plasma membrane H^+^-ATPase could promote the resistance of plum seedlings to osmotic stress. The notion was in accord with the results of previous research (Michelet et al., 1994). More interestingly, we found that the osmotic stress-induced increase of the enzyme activity was coupled with the increases of four forms of conjugated PAs, non-CC Spd (Figure 5B), non-CC Spm (Figure 5C), CC Put (Figure 6A), and Spd (Figure 6B) in the plasma membrane of the drought-tolerant plum cultivar roots. Furthermore, under osmotic stress coupled with the application of exogenous Spd, Spm, MGBG, or o-phenanthroline, the changes in the levels of four conjugated PAs were in parallel with the altering in the enzyme activity. Plasma membranes host hundreds of transport proteins, some of which are known to be strongly affected by PAs (Janicka-Russak et al., 2010). For example, Williams (1997) suggested that ion channels were regulated by conjugated polyamines. Additionally, Liu et al. (2000) reported that inward rectifying K^+^ channels in guard cells are suppressed by endogenous PAs, which promote stomatal closure under drought stress. Dobrovinskaya et al. (1999) argued that PAs could inhibit the ion channels in the tonoplast.

In addition to the researches mentioned above, over the last two decades, a bulk of significant and interesting data was accumulated providing explicit evidence for polyamines playing an essential role in regulating plant membrane transporters. The most straightforward example is a blockage of the two major vacuolar cation channels by polyamines, indicating the PA effect is direct (Pottosin and Shabala, 2014). On the contrary, in pea roots, exogenous Spm caused net H^+^ influx, and Put caused net H^+^ efflux by activating Ca^2+^ pumping across the root epidermis, regarded as a signaling pathway induced by PAs. Additionally, Spm but not Put caused a direct inhibition of H^+^ pumping in isolated plasma membrane vesicles (Pottosin et al., 2014). These results showed that the effects of polyamines on the plasma membrane cation channels are hardly dependent on polyamine species, indicating the PA effect is likely indirect (Pottosin and Shabala, 2014; Pottosin et al., 2021). Zarza et al. (2020) report that PAs can induce a rapid increase in PIP_2_ in *Arabidopsis* seedlings, triggering a massive K^+^ efflux. Thus, the causal role of PA in plant adaptive responses to the environment may be established only in the strict context of the tissue- and organelle-specificity (Janicka-Russak et al., 2010; Pottosin and Shabala, 2014).

There is another pathway, by which PAs modulate H^+^-ATPase in the plasma membrane, as mentioned in the “Introduction” of the paper. Spd and Spm could be conjugated non-covalently to 14-3-3 protein loop 8 more easily than Put because they carried more positive charges (Garufi et al., 2007). 14-3-3 protein activated by conjugated Spd and Spm could bind to the auto-inhibitory domain in H^+^-ATPase to activate the enzyme (Svennelid et al., 1999; Jelich et al., 2001; Athwal and Huber, 2002; Falhof et al., 2016). So in the research, non-CC Spd and non-CC Spm, rather than non-CC Put, functioned in regulating the enzyme activity. Of course, non-CC Spd and non-CC Spm in plasma membrane could affect plasma membrane physical state to regulate the enzyme activity (Zhang et al., 2002). The notion was in good agreement with our results. In this research, according to the effects of exogenous Spd or Spm application on RPMP and MDA contents of drought-sensitive plum cultivar under osmotic stress, and effects of inhibitors MGBG or o-phenanthroline on the two indexes of tolerant cultivar, we could infer that conjugated PAs might stabilize plasma membrane structure. Regarding the significance of CC Put and Spd, with the previous report (Del Duca et al., 1995), we concluded CC Put and Spd could maintain H^+^-ATPase structurally and functionally under osmotic stress by conjugating covalently to the Glu residues of plasma membrane H^+^-ATPase.

In addition to the regulation modes mentioned above, the observed variations in H^+^-ATPase activity in this research and its modulation by PAs might also result from the changes at the level of gene expression (Pottosin et al., 2021). Since that omic tools are significant to understanding the biological network of abiotic stress responses, prospective work using omics approaches should be essential to further understand the molecular machinery, by which PAs regulate H^+^-ATPase activity (Ebeed et al., 2019).

## Conclusion

In summary, the present work was the first to illuminate that polyamines conjugated to plasma membrane could enhance the tolerance of plum seedlings against osmotic stress by stabilizing membrane structure and therefore elevating H^+^-ATPase activity. On the theoretical side, our findings revealed one novel mechanism underlining PA-mediated osmotic stress tolerance. On the side of practical application, the levels of PAs conjugated to the plasma membrane in roots might be considered as markers of plum seedling tolerance to drought stress, and enhancing plum seedling tolerance with the proper concentration of exogenous Spd or Spm would help broaden PA practical application in forestry. The strong positive relationship between abiotic stress and polyamines has been proposed as a potential marker of environmental stress in forest trees in which phenotypic symptoms of stress are not yet visible. The markers might help forewarn forest managers to undertake amelioration strategies before the appearance of visual symptoms of stress and damage (Minocha et al., 2014).

## Data Availability Statement

The raw data supporting the conclusions of this article will be made available by the authors, without undue reservation.

## Author Contributions

HL conceived the project and reviewed the manuscripts. HD and BC performed the experiments, carried out the data analyses, and wrote the original draft. QL and RK reviewed the manuscripts. All authors contributed to the article and approved the submitted version.

## Conflict of Interest

The authors declare that the research was conducted in the absence of any commercial or financial relationships that could be construed as a potential conflict of interest.

## Publisher’s Note

All claims expressed in this article are solely those of the authors and do not necessarily represent those of their affiliated organizations, or those of the publisher, the editors and the reviewers. Any product that may be evaluated in this article, or claim that may be made by its manufacturer, is not guaranteed or endorsed by the publisher.
